# Supplementary material for: Health insurance, healthcare utilization and language use among populations who experience risk for tuberculosis, California 2014–2017
Source: PLoS One. 2022 May 24;17(5):e0268739. doi: 10.1371/journal.pone.0268739 (PMC9129044; doi:10.1371/journal.pone.0268739)
Supplement: S1 Table — (DOCX) [file pone.0268739.s001.docx]

**Supporting information**

S1 Table. Proportion language spoken in medical visit by country of birth, California 2014-2017

| Language | United States | Philippines | Vietnam | China | India | Korea | Mexico | Other Country |
| --- | --- | --- | --- | --- | --- | --- | --- | --- |
|  |  |  |  |  |  |  |  |  |
| English | 99 (99-99) | 91 (87-95) | 41 (31-50) | 52 (45-60) | 96 (94-99) | 52 (41-63) | 38 (36-41) | 76 (72-79) |
| Tagalog |  | 8 (4-12) |  |  |  |  |  |  |
| Vietnamese |  |  | 55 (46-63) |  |  |  |  |  |
| Mandarin |  |  | 2 (0-7)** | 30 (23-36) |  |  |  | 2 (1-3) |
| Cantonese |  |  |  | 17 (11-22) |  |  |  |  |
| Asian Indian Languages |  |  |  |  | 3 (1-6)* |  |  |  |
| Korean |  |  |  |  |  | 47 (36-59) |  |  |
| Spanish |  |  |  |  |  |  | 60 (58-63) | 20 (17-23) |

Source: California Health Interview Survey, 2014-2017.

Notes: * Statistically unstable - Coefficient of Variation > 0.3
