# Supplementary material for: Health insurance, healthcare utilization and language use among populations who experience risk for tuberculosis, California 2014–2017
Source: PLoS One. 2022 May 24;17(5):e0268739. doi: 10.1371/journal.pone.0268739 (PMC9129044; doi:10.1371/journal.pone.0268739)
Supplement: Attachment — Submitted filename: PLOS ONE - revisions and responses v2.docx [file pone.0268739.s002.docx]

Response to editor’s and reviewers’ comments:

Editor

1. Please ensure that your manuscript meets PLOS ONE's style requirements, including those for file naming. The PLOS ONE style templates can be found at

 DONE.

2. In your Methods section, please provide additional information about the analysis performed, for example how many participants were included.

DONE. Methods now include the line: “Data were based on respondent self-report and analysis was limited to persons 18 years and older (n=82,758).”

3. Please consider modifying your title to ensure that it is specific and descriptive.

DONE. The manuscript is now titled “Demographics and healthcare utilization of populations at risk for tuberculosis, California 2014-2017.”

4. We note that you have indicated that data from this study are available upon request. PLOS only allows data to be available upon request if there are legal or ethical restrictions on sharing data publicly. For information on unacceptable data access restrictions, please see [http://journals.plos.org/plosone/s/data-availability#loc-unacceptable-data-access-restrictions](https://urldefense.com/v3/__http:/journals.plos.org/plosone/s/data-availability*loc-unacceptable-data-access-restrictions__;Iw!!AvL6XA!kLKeEomE-iHy3BtI_DHgtkBTnPduDGRLUOUN5bE9t8s3irlSqQT_MTAxyrp3gIqqhqxk0ds$).

In your revised cover letter, please address the following prompts:

4a) If there are ethical or legal restrictions on sharing a de-identified data set, please explain them in detail (e.g., data contain potentially identifying or sensitive patient information) and who has imposed them (e.g., an ethics committee). Please also provide contact information for a data access committee, ethics committee, or other institutional body to which data requests may be sent.

DONE. Addressed in cover letter.

5. Your ethics statement should only appear in the Methods section of your manuscript. If your ethics statement is written in any section besides the Methods, please move it to the Methods section and delete it from any other section. Please ensure that your ethics statement is included in your manuscript, as the ethics statement entered into the online submission form will not be published alongside your manuscript.

**DONE.** Ethics statement now included in methods section and deleted from other sections.

6. Please include your tables as part of your main manuscript and remove the individual files. Please note that supplementary tables (should remain/ be uploaded) as separate "supporting information" files

**DONE.** Tables are now included as part of main manuscript file.

Reviewers' comments

Reviewer #1: It would be nice to have input from your populations of interest (foreign born people) – either to put in this paper, or as future steps as you engage with providers and patients at risk.

Revised to address comment: “We envision these efforts will be multi-faceted, multi-sectorial, and adapted to specific communities and providers and that they include input from populations at risk for TB”

Also, what is currently being done in terms of outreach to specific communities? What have other states done?

Added the following text to the introduction: “In California and elsewhere, public health programs are engaging with community groups especially those focused on healthcare and representing affected communities…”

Nice to have more CA specific background stats on TB and more specifically immigrant populations – what makes CA different/better/worse than other states?

Added CA background statistics to the introduction: “California bears a disproportionate share of the TB burden in the U.S. In 2019, there were 2,113 TB cases in California, 24% of all cases nationally despite having 12% of the U.S. population [4, 5]. Of Californian TB cases, 84% occur among persons born outside the United States and rates of tuberculosis among non-US-born Asians are 50 times that among US-born whites [6].”

Not sure why looking at attitudes/feelings around discrimination – while interesting, they don’t really add to the efforts/next steps around TB prevention (or this wasn’t made clear in the paper)

Revised manuscript to underscore the connection between racial discrimination and TB prevention activities. “Barriers to healthcare access and utilization, such as cost and racial/ethnic discrimination, affected less than one in ten persons. Racial or ethnic discrimination has been shown to reduce access care and filling of prescriptions which would hamper TB prevention activities [26, 27].”

Line 230 – not clear what % of homeless are foreign born. They couldn’t be surveyed, so how do we know they are not a large %?

Important point. We added a section in the limitations to address this issue: “Because this analysis focuses on non-U.S.-born persons and persons experiencing homelessness maybe under-represented, it would be helpful to know the proportion of non-U.S.-born persons who experience homelessness in California. Unfortunately, there is no statewide estimate for non-U.S.-born homelessness, but a recent study found the proportion of lifetime homelessness among non-U.S.-born persons was 1% [28].”

Need to clearly elucidate next steps with regard to improving EMR documentation of country of birth. Is there anything that can be included in the next statewide survey to help this TB campaign?

Agreed. Added this language to the discussion of EMRs: “EMRs should be modified to accommodate country of birth data and workflows should be modified to collect these data, without which a large portion of the at-risk population could be missed.”

We continue to work with partners to include TB questions on the statewide survey though to date questions on TB have not been included.

Good point about leveraging comorbidity work (smoking/diabetes).

Agreed. Helpful to know this made sense to the reviewer.

It seems like different countries at birth (e.g. Mexico) have different needs – maybe focus on some of those. County of birth being Mexico has the largest proportion of TB cases (in Table 1, 21), and in lines 230-231 you state ¼ are uninsured – seems like a good part to focus prevention efforts on. This pop also has a high percentage of diabetes.

We have identified several populations that require increase testing and treatment. From the conclusion: “We envision these efforts will be multi-faceted, multi-sectorial, and adapted to specific communities and providers and that they include input from populations at risk for TB.”

Focus on provider education in terms of increased testing for TB in this population/routine testing (not patient – in line 232). And who are these providers caring for foreign born populations? (do they differ than other providers?)

We believe that both patient and provider awareness is important to successful intervention to increase testing and treatment. Provider engagement is discussed in the context of co-morbidities: “The prevalence of these risk factors among populations already at risk for TB provides opportunities for engagement with providers who may not currently think about TB even when seeing persons at risk for the disease. One engagement strategy could be using a common comorbidity such as diabetes as an entry point for provider education about TB.”

With regard to details on the providers who serve the population at risk, we are limited by this survey questionnaire which does not include much data on providers beyond language used at visit and the patient’s usual source of care.

What about the following factors in describing your pop of interest: legal status (esp for people interested in obtaining legal status – might be less likely to utilize any services out of fear of jeopardizing their future greencards), travel back to country of origin (perhaps this is where they get TB, thru travel, and not just coming into the US with it), age at immigration to US, household size/make up of extended family (and if they have travel back to at-risk countries, or issues of household crowding making it easier to spread TB).

We are limited by the survey questionnaire which does not include data on travel to country of origin.

Would be nice know breakdown of active TB cases (foreign born vs. US born), does this follow the same patterns as described in this paper?

Added the following to the introduction: “California bears a disproportionate share of the TB burden in the U.S. In 2019, there were 2,113 TB cases in California, 24% of all cases nationally despite having 12% of the U.S. population [4, 5]. Of Californian TB cases, 84% occur among persons born outside the United States and rates of tuberculosis among non-US-born Asians are 50 times that among US-born whites [6].”

Table 1 – more “TB Burden” to be at top of table, not at bottom

Revised per comment.

Table 4 – include percentages

Other reviewers also commented on this table. I have revised the table to be more understandable. Percentages are included.

Reviewer #2: Overall, I found this an interesting analysis of the demographic, health, and healthcare seeking characteristics of the non-US born population in California. While this population is certainly important to identify, describe, and understand for TB control, the paper lacks a clearly presented conceptual framework that describes how these demographic and clinical characteristics: 1) impact TB care and outcomes; and 2) are unique to TB.

- Re-wrote introduction to address lack of conceptual framework and to describe how healthcare utilization is important in planning TB prevention activities. Essentially, TB prevention activities rely on access and use of healthcare. One of the main interventions is to have persons at-risk for TB be tested by their doctor and treated if needed.

The non-USB population is at risk for a variety of conditions (Hepatitis, COViD, e.g.) making it important to clearly describe the context for focus on non-USB more explicitly in the introduction.

- Add context for the focus on non-USB in introduction: “California bears a disproportionate share of the TB burden in the U.S. In 2019, there were 2,113 TB cases in California, 24% of all cases nationally despite having 12% of the U.S. population [4, 5]. Of Californian TB cases, 84% occur among persons born outside the United States and rates of tuberculosis among non-US-born Asians are 50 times that among US-born whites [6].”

A conceptual framework for why each of these data fields from CHIS were chose would also be helpful, especially for readers not as familiar with the domestic TB literature.

- Revised to clarify connection between health utilization and tuberculosis : “To make progress against TB in California, the number of persons that are tested and treated for LTBI needs to be substantially increased. Recent mathematical models support the scale-up of targeted testing and treatment of non-U.S.-born persons for TB infection as important for the reduction of TB in California [8, 9]. However, these TB prevention activities rely on access and use of healthcare and our knowledge of these attributes among non-U.S.-born persons is incomplete. In California and elsewhere, public health programs are engaging with community groups especially those focused on healthcare and representing affected communities but more information is needed about the demographics, healthcare utilization and potential barriers to care of populations at-risk for TB [10, 11]. Recent studies have highlighted disparities in healthcare utilization among minority groups but have not focused on non-U.S.-born persons at-risk for TB [12, 13]. Understanding healthcare utilization of at-risk populations is important for the planning of TB prevention activities.

Please specify in Methods why you focus on 5 countries named (assuming based on country of origin for most TB cases in CA?).

We revised the methods section to address this comment: “We calculated survey proportions and 95% confidence intervals (CI) stratified by country of birth, focusing on the six countries with the highest number of TB cases in California, specifically Mexico, United States, Philippines, Vietnam, China, and India. These countries accounted for 80% of all cases in California during 2016-2017 [18, 19].”

Suggest a citation for this statement as it forms a key underlying premise for the purpose of the paper (ln 67)” One specific barrier to implementing TB prevention in large health systems is that country of 68 birth, the most important risk factor for TB, is often not captured in the electronic medical 69 record (EMR).”

Citation added.

This statement does not seem to make sense (ln 277-278) “Our results show that non-English language at medical visit was an excellent proxy for non-U.S. 278 birth, but identified only half of all non-U.S.-born persons.” How is a proxy excellent if it only identifies half of the eligible population? Please refer to the 2-way table you present in results as to how you would like the reader to interpret this statement. Are you say that the positive predictive value or specificity is high but sensitivity is low? If so, please state as such.

Agreed. We have revised the language toclarify: “Fifth, persons who used a language other than English at their medical visit were highly likely to be born outside the U.S. Thus, preferred language may be suitable as a starting point for identifying high-risk subgroups. However, non-English language at medical visit identified only half of all non-U.S.-born persons.”

We also mention high specificity and low sensitivity in the results section: “As a proxy for nativity, language at medical visit had a low sensitivity (42%) and a high specificity (99%) (table 3).” Please note the table numbering has changed for tables 3 and 4.

Please describe important limitations of using CHIS data, ie, sampling bias based on phone and self-response and how this may actually underestimate the prevalence of some of those key sociodemographic determinants that affect TB care.

Agreed. The following text was added to the limitation section: “The survey was conducted by phone and may under-represent persons who are less likely to answer the phone, such as persons experiencing homelessness, young adults, and non-English speakers. Furthermore, it relies on participant self-report which may underestimate key determinants of TB risk, such as poverty and country of origin, because of social desirability bias.”

Table 3 is challenging to read and interpret. Suggest revising.

Agreed. We have simplified the table by splitting it into two parts and relegating one part to the supplement. The table is now table 4 and Supplement Table 1.

You may want to consider using the first paragraph of the Discussion as the last paragraph of the Introduction, as it is the first place that seems to clearly articulate the purpose for the analysis presented.

Yes, very helpful suggestion. We have moved the first paragraph of the discussion to the end of the introduction.

Reviewer #3:

This is an interesting paper. It has the potential benefits in the field of TB in the USA. But to let it be strong enough, some clarifications are needed. Below are some suggestions which could help improve the paper if the authors found them relevant.

- I would suggest to further explain the methodology. I’m a little bit concerned with the methodology section as presented. The methods section should be clearly and unambiguously stated. I find that there are many references that are indicated and that unfortunately hide a lot of details. For example, the methodology does not show the countries or let's say the different nationalities (India, Mexico, USA, Philippine, Vietnam, etc.) mentioned in the results section. Is there any link or relationship between individuals regarding their nationalities and the risk to get TB? Also, what is the threshold and the criteria of poverty according to the place of birth? According to the fact that poverty in the US could be completely different of the one in Philippine or somewhere else…

We have revised the methods section to address these concerns. With regard to countries of origin we have included the following text: “We calculated survey proportions and 95% confidence intervals (CI) stratified by country of birth, focusing on the six countries with the highest number of TB cases in California, specifically Mexico, United States, Philippines, Vietnam, China, and India. These countries accounted for 62% of all cases in California during 2016-2017 [14, 15].”

In reference to poverty, we added this language: “Poverty was defined as less than 139% of the U.S. federal poverty level. The respondent’s percentage of federal poverty level was calculated using household income earned in the U.S. and household size.”

- HMO. I don’t know what this acronym means! Please put the definition in bracket (p.99)

Apologies for this oversight! We have included the full wording now in the methods section: “Usual source of care was condensed from seven categories to three as follows: doctor’s office, health maintenance organization (HMO) or Kaiser were grouped as doctor’s office…”

- In most of the items presented in the results section, it is not easy to perceive the link between the different factors (diabetes, smoking, good or bad health, etc.) mentioned and their relationship with TB. The authors should discuss this further.

Revised the discussion to clarify the link between risk factors and tuberculosis. Here’s the passage from the discussion section: “Third, risk factors for the progression of latent TB infection to TB disease varied widely among the populations examined. Diabetes and smoking are associated with increased risk of progression of latent TB infection to active TB disease [23, 24]. Self-reported diabetes among Philippines-born and Mexican-born persons was more than double the rates for persons born in other countries including the U.S., similar to prior work [25, 26]. Likewise, former smoking was twice as high among Philippines-born and Mexican-born persons than among persons born in China, India or Vietnam as has been previously documented [27, 28]. The prevalence of these risk factors among populations already at risk for TB provides opportunities for engagement with providers who may not currently think about TB even when seeing persons at risk for the disease.” We also add the following text to the methods section “Smoking and diabetes were considered because they are risk factors for progression of latent TB infection to TB disease [20, 21].”

- The discussion section, as presented, in my opinion is more a part where the authors interpret their results and not discussing them. The discussion, as its name indicates, should be the place where the results, while being interpreted, are also confronted with the studies of other authors and where the points of view are clearly confirmed, refuted or nuanced. And this should be felt in the richness of the references mentioned in the said section. But, as we can see here, the authors have mentioned only 2 references in this whole long discussion... And besides, the article is not rich enough in terms of bibliographic references. So, I suggest to enrich it further, if only with a little more reading...

We a revised the discussion to juxtapose our results with results from the literature and have now included nine references in this section.

- Where is the conclusion section? And what are the main conclusions of the data obtained from the California Health Interview survey (CHIS)?

We have added a conclusion section: “Our analysis points out several important differences in demography, healthcare access and utilization, and language use among populations at risk for TB by country of birth. These differences present opportunities and signal potential pitfalls for planning outreach to specific communities at risk for TB and the providers, clinic systems, health insurers and health insurance purchasers who serve them.”

- What could be the concrete added value of this article? What contribution and especially how can the results obtained contribute to the elimination of TB in the whole USA in general or at least in California more specifically? This is not clear in the paper.

We have include extra language on the concrete value added of this article in the conclusion: “These data could be used to inform public awareness campaigns, provider education, academic detailing, and community outreach efforts. We envision these efforts will be multi-faceted, multi-sectorial, and adapted to specific communities and providers and that they include input from populations at risk for TB. While the information here most directly supports this tailored approach in California, we believe that similarly crafted approaches would help elimination TB in other states and across the U.S.”
