# Supplementary material for: Health insurance, healthcare utilization and language use among populations who experience risk for tuberculosis, California 2014–2017
Source: PLoS One. 2022 May 24;17(5):e0268739. doi: 10.1371/journal.pone.0268739 (PMC9129044; doi:10.1371/journal.pone.0268739)
Supplement: Attachment — Submitted filename: Response to reviewers.docx [file pone.0268739.s004.docx]

Second Revision - Response to Reviewers:

***************************************************************************

Reviewer #4: All comments are included in the attachment review report, the authors are particularly recommended to give adequate attention revising the manuscript for coherence of ideas and some areas need further clarification. For instance abstract.

We have complete extensive revisions to improve the coherence of ideas and clarify areas of the manuscript that were muddled, especially the abstract. I have included the point by point comments and responses here, but it may be more convenient for the reviewer to view them in context in the track changes version of the manuscript which has been uploaded as part of this submission.

Title: “unncessary authors can discuss about demographics under the health care utilization title-----"

Revised per request. Modified text to avoid stigmatizing language

Title: “I have searched to know who are popln at risk but nowhere the authors deined “

Very good point. I have included the definition in the methods section (line 109) and revised the introduction to highlight these groups (line 58).

Comment on short title: “not sure whether this is important”

Submissions require short title under 100 characters

Comment on abstract methods: “very much shallow make it has to be detail”

Made revisions here to provide more detail on the methods.

Comment on abstract methods language which recapitulated old mansucript title: “not clear how relevant”

Deleted reference to language use here. The relevance is discussed in the manuscript

Comment on abstract conclusion: “better conculusion”

Revised the conclusion to highlight an important finding which was that populations who experience risk for TB had health insurance and used healthcare.

Line 100: “why?”

Modified language to explain why adult survey was used.

Methods, Line 104: “very large pargraph better to make short and clear paragraphs”

moved to appropriate subsection below.

Line it is better if the authors descibe each methodology scetion separately

Agreed. Added subsection headers and re-arranged text to fit into subsections.

Methods: “this seems operational definition of terms,I think authors need to have operational definition section”

Agreed. Add subsection headers and re-arranged text to fit into subsections.

Results: “I think authors are not following plos one author guide for writing tables and graphs”

I have reviewed the table requirements and I believe that all tables in this document comply with the author guidelines. Can the reviewer or editor please specify what about the tables does not follow journal style guidelines?

Results section, health insurance subsection: “how do authors corrplate health insurance with utilization of health service;healthcare financing vs utilization. is it this is not out of the scope of this study”

The focus of the paper is on the differences in health utilization between populations who experience risk for TB. Health insurance is a key component of healthcare access, but alone it does not ensure utilization. That is why we looked at both health insurance and healthcare utilization. Again, our interest was to _­_understand if these populations had different insurance or utilization levels.

Results section, Overall Health and Risk factors for TB reactivation subsection: “journal requirment”

As above, apologies, I am unclear as to what does not meet the journal style requirement here. Can you specify what I can do to bring this in to compliance with journal style guidelines?

Table 3: “such descriptions are expected in testing new tests and gold standard test in epidemiology why authors included here, not clear”

In the introduction, we discussed that country of birth, a key risk factor for TB, was missing in many EHRs but that preferred language was used as a proxy. Here, we are setting up results for the use of language at medical visit as a method for identifying persons born outside the U.S. We thought that the standard calculations on sensitivity and specificity would be help readers understand how language could be used to identify non-U.S.-born. We draw conclusions from these results in the discussion (line 341). We have added language in discussion to further clarify the use of language as a proxy.

Discussion: “what will be the recommendation of the authors for this result;aganist health insurance”

Apologies, I’m not sure I fully understand the comment. The result that most persons who experienced risk for TB had health insurance is a positive one, in that, it suggests that having health insurance is not a barrier to accessing healthcare for many in these populations. We highlight the importance in line 291 “With the notable exceptions of persons experiencing homelessness and one quarter of persons born in Mexico who are uninsured, the hurdle of getting at-risk populations into care has largely been met.” We are certainly not recommending against health insurance. Perhaps the reviewer could further explain?

Acknowledgements: “many of the jouranl requrements are not fullfilled”

As above, apologies, but after reading through the journal requirements again, I am unsure as to what about the tables and table references is not in compliance with journal requirements. Could you specify what in particular about the tables and table references does not meet journal requirements?
